# Adaptive switch to sexually dimorphic movements by partner-seeking termites

**DOI:** 10.1126/sciadv.aau6108

**Published:** 2019-06-19

**Authors:** Nobuaki Mizumoto, Shigeto Dobata

**Affiliations:** 1 Laboratory of Insect Ecology, Graduate School of Agriculture, Kyoto University, Kitashirakawa-oiwakecho, Sakyo-ku, Kyoto 606-8502, Japan.; 2 School of Life Sciences, Arizona State University, ISTB1, 423, East Mall, Tempe, AZ 85287, USA.

## Abstract

How should females and males move to search for partners whose exact location is unknown? Theory predicts that the answer depends on what they know about where targets can be found, raising the question of how actual animals update their mate search patterns to increase encounter probability when conditions change. Here, we show that termites adaptively alternate between sexually monomorphic and dimorphic movements during mate search. When the location of potential mates was completely unpredictable, both sexes moved in straight lines to explore widely. In contrast, when the stray partner was at least nearby, males moved while females paused. Data-based simulations confirmed that these movements increase the rate of successful encounters. The context-dependent switch of search modes is a key to enhance random encounters.

## Teaser

The key to successful pairing of termites is alternation between different movement modes depending on informational context.

## INTRODUCTION

Moving for survival and reproduction is nearly a defining characteristic of animals (*1*–*3*). Their movement patterns to find targets depend on the availability of sensory information and/or memories about targets (*4*, *5*). When searching for targets with little or no locational information, animals adopt random walks (*6*, *7*). A number of theoretical studies have collectively revealed that the optimal movement patterns for random walks depend on the searching conditions, including density, distribution, and movement of targets (*4*, *5*, *8*–*13*). Therefore, the detailed conditions must be specified to accurately evaluate the efficiency of movements observed in animals (*5*, *14*). Some studies have tried to test how animals adaptively search for targets by focusing on marked changes of search modes depending on environmental conditions (*15*–*17*). However, as the search contexts are rarely exactly specified, most predictions remain empirically untested.

**Fig. 1:**
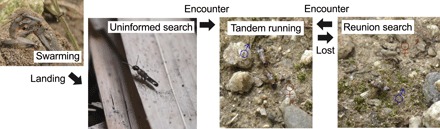
Mating biology of subterranean termites, illustrated by *R. speratus*. In the mating season, alates (winged adults) fly off in large swarms to disperse. After dispersing, individuals shed their wings and walk to search for a mating partner without any previous locational information. Successfully encountered pairs then run in tandem to search for a suitable nest site, with the male following the female. Tandem running is quite synchronized, where a pair moves like a single individual. Nevertheless, they are sometimes separated and must search to find each other again. Thus, there are two search situations with different uncertainty about the location of their partner (before pair formation: uninformed search; after separation: reunion search). Photo credit: Nobuaki Mizumoto, Arizona State University.

Mating is one of the main motivations of search. Optimal mate search movements are sometimes sexually dimorphic as a result of mutual evolutionary optimization (*18*–*20*). In monogamous mating systems, in which each individual mates with only one partner, theory predicts that the degree of sexual dimorphism in search strategies should depend on the potential distance to a partner; if partners are completely randomly positioned, both males and females are expected to move in a straight line (*21*), but if there is a specific available search time and expected distance to a (potential) partner, then the optimal movement patterns can be sexually dimorphic (*19*). To test this prediction, we focused on the mating biology of subterranean termites (Rhinotermitidae). These termites face the challenge of finding a mating partner under a variety of uncertain conditions. During a brief period of the year, alates (winged adults) fly out of their nests and disperse by wind (Fig. 1). After dispersing, both males and females land on the ground, shed their wings, and run to search for a mating partner (Fig. 1) (*22*, *23*). In this process, dealates (wing-shed adults) should have no idea of the surrounding environment because they come out of their nests for the first time. Moreover, their mate search behavior is poorly informed because the weak pairing pheromones emitted by females are effective only within a few centimeters or on contact (*24*). Upon joining successfully, a pair performs tandem running in which the male follows the female by maintaining almost constant contact with her back in a highly coordinated manner (Fig. 1) (*25*). They seek a suitable site to establish their nest, where they form a lifelong monogamous royal pair.

There are two situations where females and males have to manage uncertainty about the location of their partner during the mating process. First is when they completely lack locational information of a potential partner before pair formation. Second is when the pair is accidentally separated after pair formation (*22*). In the latter case, they know that the partner must be at least nearby, although the exact location is uncertain (Fig. 1; see also Supplementary Text and fig. S1). We hypothesized that termite dealates alter their movement patterns depending on the two situations described above and thereby promote the efficiency of partner search.

We investigated the movement patterns of dealates of two termite species, *Reticulitermes speratus* and *Coptotermes formosanus*, focusing on sex differences during mate search. Both of these species show a typical sequence of mate search and monogamous colony foundation. We first observed the searching behavior of dealates before pair formation and then added a mating partner to induce tandem running. Then, we removed the mating partner from the tandem to observe the change in their searching behavior after separation (movies S1 and S2). Their motion was characterized by movement speed, turning pattern, and behavioral intermittency (pause-move patterns) as the main elements of a random walk (*10*). Using parameter values estimated from observations, we simulated their movement patterns under the respective searching situations to evaluate their efficiency.

## RESULTS

### Quantitative movement analysis of partner search by termites

**Fig. 2:**
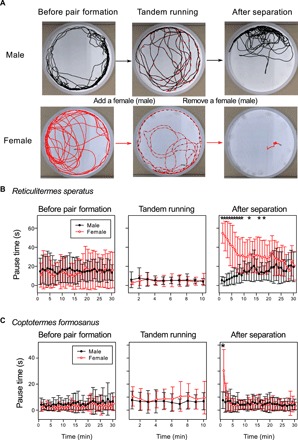
Pause behaviors of termite dealates across different periods of mate search. We first observed searching behaviors before pair formation. Then, we added a single mating partner of the other sex to observe their movements during tandem running. Last, we carefully removed the partner using an aspirator and observed how they change their searching behaviors. (**A**) Representative trajectories of dealates of *R. speratus* for two different trials, that is, focusing on a male (above) and a female (below). Trajectories are for 5 min for before pair formation and after separation, while they are for 3 min for tandem running. The movement trajectories of the added partner (above, female; below, male) were shown in dashed lines. Before pair formation, males and females showed similar movements, whereas they showed distinct sexually dimorphic movements after separation (movie S1). (**B** and **C**) Comparison of pause time between sexes in (B) *R. speratus* and (C) *C. formosanus*. Both species showed sexually dimorphic movements immediately after separation, where females often paused and males moved actively. Points with bars represent mean values with SDs. “*” indicates significant differences between sexes (Wilcoxon rank sum test with Bonferroni corrections, *P* < 0.05/70). Photo Credit: Nobuaki Mizumoto, Arizona State University.

Overall, the movement patterns of both *R. speratus* and *C. formosanus* were sexually monomorphic before pair formation as well as during tandem running after pair formation, whereas they showed remarkable sexual dimorphism when separated after pair formation (Fig. 2). Before pair formation, search was characterized by active movements irrespective of sex. The standard deviations of movement components overlapped between sexes, including pause duration (Fig. 2, B and C), movement speed (fig. S2), and turning angle (fig. S3) (see Materials and Methods). During tandem running, movements of the dealates were also sexually monomorphic but with small differences among pairs (Fig. 2 and figs. S2 and S3). In stark contrast, the termites showed distinct sexually dimorphic movements immediately after separation: Females paused for long periods, while males paused only briefly and moved actively (Fig. 2 and movies S1 and S2). Over time, their movements gradually returned to the state before pair formation (Fig. 2 and figs. S2 and S3). This sexual dimorphism was observed for a longer period in *R. speratus* (Fig. 2B) than in *C. formosanus* (Fig. 2C). *R. speratus* also showed a sexual difference in the moving speed (fig. S2A) but not in the turning angle (fig. S3A), while *C. formosanus* showed sexual differences in both (figs. S2B and S3B).

To evaluate the searching efficiency of the observed behaviors, we parameterized their movement patterns by decomposing them into movement speeds, turning patterns, and pause-move patterns. As sexual dimorphism was the most prominent during the first minute after separation, we extracted parameters of movement patterns from data covering 1 min each before pair formation and after separation in *R. speratus* and *C. formosanus*. We used correlated random walks (CRWs) as the movement framework to measure differences of termite searching (*8*, *26*). CRW is described by two parameters: the speed and the sinuosity (see Materials and Methods). Table 1 summarizes the parameter estimates. Before pair formation, there were no statistically significant sexual differences for speed and sinuosity in either species (Table 1, corresponding to the sexual comparison at the last 1 min before pair formation in figs. S2 and S3). After separation, we detected significant sexual differences for speed in *R. speratus* (Table 1A, corresponding to the sexual difference at first 1 min after separation in fig. S2A) and for both speed and sinuosity in *C. formosanus* (Table 1B, corresponding to the sexual difference at first 1 min after separation in figs. S2B and S3B). The changes of parameter values were measured for both sexes. In both species, females slowed their movements; in addition, males of both species and females of *C. formosanus* shifted to more sinuous CRWs after separation (Table 1).

**Table 1:** Parameters extracted from movement patterns of termites and comparison between sexes and searching schemes. The sinuosity corresponds to the scale parameter of wrapped Cauchy distributions, covering from 0 (most sinuous) to 1 (straight motion). In statistics, *W* corresponds to Wilcoxon rank sum test, where parameters were compared between sexes in each scheme and species, while *V* corresponds to Wilcoxon signed-rank test, where parameters were compared between before pair formation and after separation. *P* is the *P* value, and significant results are in bold. M and F indicate male and female, where the numbers in parentheses indicate sample size. The sample size of females in *R. speratus* is variable between before pair formation and after separation because some individuals only paused during analyzed duration after separation but moved before pair formation.

**A. *R. speratus***										
**Speed**		**Mean ± SE (mm/s)**		**Wilcoxon rank** **sum test**		**Sinuosity**	**Mean ± SE**		**Wilcoxon rank** **sum test**	
		**M (19)**	**F (14, 16)**	***W***	***P***		**M (19)**	**F (13, 16)**	***W***	***P***
**Scheme**	Before	14.7 ± 0.6	14.8 ± 0.7	154	0.961	Before	0.85 ± 0.01	0.86 ± 0.01	189	0.385
	After	15.0 ± 0.8	5.4 ± 0.3	0	**<0.0001**	After	0.77 ± 0.01	0.77 ± 0.06	154	0.461
**Wilcoxon** **signed-rank** **test**	*V*	106	0			*V*	9	29		
	*P*	0.679	**0.0001**			*P*	**0.0001**	0.2734		
B. ***C. formosanus***										
**Speed**		**Mean ± SE (mm/s)**		**Wilcoxon rank** **sum test**		**Sinuosity**	**Mean ± SE**		**Wilcoxon rank sum test**	
		**M (22)**	**F (20)**	***W***	***P***		**M (22)**	**F (20)**	***W***	***P***
**Scheme**	Before	20.7 ± 1.4	22.3 ± 0.9	279	0.142	Before	0.84 ± 0.01	0.85 ± 0.01	274	0.18
	After	19.0 ± 1.2	10.1 ± 1.1	43	**<0.0001**	After	0.79 ± 0.01	0.82 ± 0.01	310	**0.0231**
**Wilcoxon** **signed-rank** **test**	*V*	104	0			*V*	15	35		
	*P*	0.4826	**<0.0001**			*P*	**<0.0001**	**0.007**		

In both species, females paused for more than a half of the period, while females before pair formation and males showed pausing behaviors with short periods (fig. S4). We analyzed move-pause patterns of termites across searching schemes and sexes (see Materials and Methods), because the move-pause intermittency is considered as an elemental component of movement patterns (*10*, *27*); the durations of moves and pauses often follow power-law distributions (*28*, *29*). When we fitted truncated power-law and stretched exponential distributions to the data, maximum likelihood estimations and Akaike weights indicated that truncated power-law distributions better described the pause durations except that of male *R. speratus* before pair formation (fig. S5 and table S2), whereas the move durations of male *R. speratus* and of both females and males of *C. formosanus* before pair formation were better fitted to stretched exponential distributions (fig. S5 and table S2).

These pausing behaviors are closely related to turning behaviors, because when animals finish pausing behaviors, they tend to choose new directions that might be less correlated with the previous ones, which is called reorientation behaviors (*10*). Therefore, we also analyzed the data of turning angles that occurred after pauses and during moving separately (see Materials and Methods). We found that termites, irrespective of species and sexes, showed significantly larger turning angles after pauses than during moving (table S1). Moreover, we found weak but statistically significant positive correlation between the pause durations and absolute values of turning angles in *R. speratus* (linear model; *R. speratus*: slope ± SEM = 0.039 ± 0.001, *F* = 22.31, *P* < 0.001; *C. formosanus*: *F* = 1.496, *P* = 0.222). These results suggest that termites show reorientation behaviors after pauses.

### Assessment of searching efficiency of random walks by termites

**Fig. 3:**
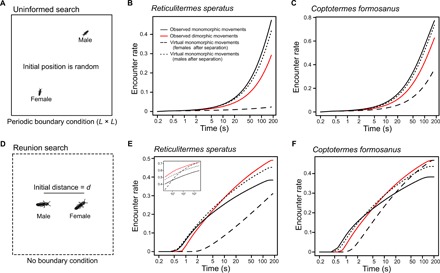
Searching efficiency of termite movements in two different searching situations. (**A**) Assumed search conditions before pair formation (uninformed search). Females and males search for partners without any locational information, which was simulated by periodic boundary conditions (size *L* × *L*) with random initial positions of females and males. (**B** and **C**) Searching efficiency under uninformed search conditions for (B) *R. speratus* (*L* = 223.6, φ = 7), where observed monomorphic movements and virtual monomorphic movements are overlapped, and (C) *C. formosanus* (*L* = 223.6, φ = 10). In both species, sexually monomorphic movements observed before pair formation achieved the higher encounter rates. (**D**) Assumed search conditions after a pair gets separated (reunion search). After separation, the distance between them is expected to be short. Such conditions were simulated by borderless continuous spaces with a distance *d* between a female and a male. (**E** and **F**) Searching efficiency of observed movement patterns under reunion search conditions for (E) *R. speratus* (*d* = 16.09, φ = 7), where the result after 180 s is inserted (10,000 replicates and data were obtained every 20 s until 3 × 10^5^ s), and (F) *C. formosanus* (*d* = 22.97, φ = 10). In turn, sexually dimorphic movements observed after separation achieved the highest encounter rates during realistic searching period. The simulations performed by resampling empirical data to describe the durations of moves and pauses showed similar results (fig. S8). The results were obtained from means of 1,000,000 simulations.

We used simulations to test the searching efficiency of termites’ movement patterns under respective search situations (see Materials and Methods). The mate search before pair formation is the “uninformed search,” where multiple females and males search for mating partners and distances among them are completely unknown (Fig. 3A). We modeled this situation with two-dimensional periodic boundary conditions, where initial positions of a female and a male were set randomly (size = *L* × *L* mm^2^; Fig. 3A). Periodic boundary conditions can easily approximate infinite space with infinite number of individuals and are often used in models or simulations of random search problems (*4*, *9*). In our simulations, the size *L* × *L* equals to the expected area occupied by one pair of termites, which was obtained from the population density required for dealates of *R. speratus* to succeed in mating (20 pairs/m^2^) (*30*). Given this density, the expected area occupied by one pair is calculated by *L* × *L* = 120 m^2^ = 50,000 mm^2^, and thus, the size *L* was set to $\sqrt{50000}=223.6$ mm. On the other hand, the mate search after separation is the “reunion search,” where a female and a male search for their specific partner nearby without detailed locational information (Fig. 3D). This situation was modeled by a borderless continuous space, where a female and a male were initially separated by the distance *d* (Fig. 3D). To obtain the distance *d*, we observed spontaneous separations during tandem running and measured the separated distance between a male and a female every 0.2 s until they reencountered (see Supplementary Text). The distance *d* was determined to be 16.09 mm for *R. speratus* and 22.97 mm for *C. formosanus*, respectively, as the mode value of the distribution of separated distance. We evaluated four possible combinations of movement patterns: the observed sexually dimorphic movement after separation, the observed sexually monomorphic movement before pair formation, a virtual monomorphic movement where both partners moved like females after separation, and a virtual monomorphic movement where both moved like males after separation.

Under the uninformed search conditions, the observed sexually monomorphic movement always achieved higher efficiency than the observed sexually dimorphic movement and even the other virtual monomorphic combinations (*R. speratus*: Fig. 3B; C*. formosanus*: Fig. 3C). Under the reunion search conditions, the results depended on the length of search time (Fig. 3, E and F), which had been predicted in the previous theoretical study (*19*). The observed sexually dimorphic movement achieved higher efficiency than the observed sexually monomorphic movements when the search time was longer than 3.6 s in *R. speratus*, while it was longer than 5.0 s in *C. formosanus*. Within the parts of these ranges, the sexually dimorphic movement also showed higher efficiency than any other virtual combinations of movement patterns (from 9.6 to 241,840 s in *R. speratus* and from 10.6 to 187 s in *C. formosanus*). On the other hand, some sexually monomorphic combinations achieved higher encounter successes outside these ranges (Fig. 3, E and F). On the basis of our empirical observations, 91.1 and 78.6% of reunion searches for *R. speratus* (*n* = 282) and *C. formosanus* (*n* = 56), respectively, ended with reencounter within the range in which the observed sexually dimorphic movements outperformed the observed monomorphic movements (fig. S1 and Supplementary Text), illustrating that the sexually dimorphic movement was adaptive for the termites to facilitate reunion in realistic search time.

## DISCUSSION

Our simulations parameterized by empirical behavioral measurements demonstrated that the transition of different movement modes observed in termites is adaptive in promoting encounters across different informational contexts (Fig. 3), as follows. First, before pair formation, individuals have no information to locate their potential partners, and they engage actively in random search with less sinuous motions compared to those after separation (i.e., sinuosity ~ 0.85; Table 1). In random search, as animals probably find a target either nearby or faraway, they need to combine both local- and large-scale searching, where the former leads to the area-restricted search, while the latter leads to ballistic motion (*5*). In this sense, the observed movements should be an adaptive compromise of these two elements. CRWs with sinuosity similar to our results are often used by random searcher in many other animals (*31*, *32*). Second, when a mated pair gets separated after pair formation, the termites generally increase their turning rate and show remarkable sexually dimorphic movements: Females pause frequently and males move actively (Fig. 2). They have information that their mating partner must be nearby, and our simulations demonstrate that their movements are adaptive responses to reunion search (Fig. 3). This dimorphism in pausing behavior facilitates reunion: Without pausing by females, the pair would suffer from lowered encounter efficiency (Supplementary Text and fig. S6). Last, over 30 min of separation, the termites progressively change their movements from sexually dimorphic to monomorphic ones (Fig. 2). This observation suggests that this duration of searching in vain is enough for the termites to decide that the lost partner is no longer near them. Our results support the notion that animals do not engage in a one-size-fits-all search strategy but update their strategies through feedback from their experience (*5*, *15*, *33*).

A comparison between our mate search system and prey-predator systems gives an insight into flexibility of animal movements. The contrasting movements between random and reunion searches are reminiscent of previous observations that predators often alternate between extensive and intensive searches depending on prey distributions (*2*, *5*, *16*, *34*). In extensive searches, predators explore by moving for longer distances to find more profitable areas, whereas in intensive searches, they exploit prey-rich areas with frequent turns. Moreover, during reunion searches, females engaged in pausing to facilitate encounters. Similar animals that show pausing behavior in biological encounters are ambush predators, where the advantage of pausing or moving should depend on various factors such as body size and the abundance of targets (*29*, *35*). By focusing on when pausing behavior offers advantages, our results shed light on the reverse side of random search problem that asks for the most efficient way to be found by specific moving agents.

In the observed sexually dimorphic movements during reunion searches, one can regard that the male plays the role of the searcher, while the stray female plays the role of the target. In our model, the sex roles should be symmetric and interchangeable. Then, why was the pausing sex always females in termites? A reasonable explanation invokes the sexual difference in detection ranges. Tandem running of termites is often mediated by pair-bonding pheromones secreted by leading females to help males follow them (*24*). This pheromone should enable males to detect females from a longer distance than females. A previous model predicted that, with such asymmetric detection ranges, high efficiency of encounters can be achieved when the sex with a narrower detection range moves slower or even pauses (*20*). Moreover, *R. speratus* showed a larger sexual difference than *C. formosanus*, which also supports the previous model that predicts that the “short-sighted” sex should move more slowly under larger sexual differences in the detection range (*20*). The pair-bonding pheromone of *Reticulitermes* termites is volatile (*36*), whereas that of *C. formosanus* is nonvolatile and works upon contact (*37*). Therefore, *R. speratus* males can detect partner females from a longer distance than *C. formosanus* males. It should be noted that we did not observe sexually dimorphic movements before pair formation, suggesting that the mere presence of sexual difference in the detection range due to attracting signals cannot fully explain the movement patterns of termites. Integrating the effect of attracting signals and the optimal random search enabled a fuller understanding of the flexible random search strategies of termites.

An important goal in the study of optimal random search is to identify the common features of the underlying processes across species (*38*). The advantage of dimorphic movements in reunion search revealed in this study can be applied to various social interactions beyond the mate search context. For example, ants perform tandem running during nest relocation and group foraging, in which a single well-informed worker guides a naive nestmate to a target site (*39*). When the follower loses its leader, the leader pauses and waits for the missing follower, while the follower engages in a Brownian walk that is effective to search for the leader (*39*). Although form and function of tandem running are different between ants and termites, our results represent a remarkable evolutionary convergence of behavioral dimorphism that promotes encounter with a separated partner. The convergence should further be extended to our own species by asking how two people can find each other efficiently in a known search region such as the High Street or a shopping mall, known as the rendezvous search problem in operations research (*40*). This problem has many variations, and it can sometimes be optimal for one player to wait for the other (*41*). Our findings thus have potential applications to the design of human-engineered searchers (*38*). Combined with the previous studies, our results illustrate the convergence between adaptive evolution of animals and rational thinking of humans.

To conclude, biological details are essential when we evaluate the random search strategies of animals. Types of targets (*12*, *19*) and searching conditions (*11*) strongly affect the search efficiency and the resulting fitness. Comparative experiments across conditions as well as across species will offer an ideal opportunity to understand how animals search for uncertain targets in complex environments. In this study, we focused on the walking behavior of termite dealates, which has a sole function of mate search. Our data-driven modeling successfully demonstrated that the termites adaptively switch between sexually monomorphic and dimorphic movements depending on the informational contexts, which improved pairing compared to the alternative strategies. It is usually difficult for animals to achieve optimal states in a strict sense that are theoretically derived (*42*). Nevertheless, the condition-dependent changes along continuous parameters indicate the adaptive value of respective search strategies. A comprehensive view of adaptive search strategies that is achieved by clarifying their contexts will have wide applications that should contribute to our well-being, as well as deepen our understanding of life.

## MATERIALS AND METHODS

### Termites and experimental setup

We used two termite species, *R. speratus* and *C. formosanus*, which show a common sequence of mating behavior: They walk to search for mates, and the resulting pairs perform tandem running to seek potential nest sites (*23*). We collected alates of *R. speratus* from five colonies (R_A_ to R_E_) together with a piece of nesting wood from oak or Japanese cedar forests in Kyoto, Japan in May 2017, just before their swarming season. Alates of *C. formosanus* were collected using light trapping (79 individuals, C_L_) as well as from nesting wood from pine forests (two colonies, C_A_ and C_B_) in Wakayama, Japan in June 2017. All collected individuals were maintained in artificial nests at 20°C under dark conditions until the experiments to control flight timing. Just before each experiment, we transferred the nests into a room at 27°C, which promoted alates to emerge and fly. Alates were then collected and separated individually. We used individuals that shed their wings by themselves within 24 hours.

To observe mate search behavior of termite dealates, we prepared an experimental arena by filling a petri dish (φ = 145 mm) with moistened plaster so that the surface of the arena can be cleaned by slicing off plaster before each trial. A video camera (BSW50KM02BK USB2.0 camera, BUFFALO, Japan) was mounted vertically above the arena, and the camera system was adjusted so that the arena filled the camera frame. The video was recorded to a Windows PC using CCI-Pro-MR (www.cosmosoft.org/CCI-Pro-Mr/) at a resolution of 640 × 480 pixels. We extracted the coordinates of termite movements at a rate of five times per second from each video using the video-tracking system UMATracker (*43*). The arenas were illuminated by two white light-emitting diode lights with an intensity of 430 ± 60 lux. In the observation of termite behavior, each individual was used only once. All data analyses were performed using R v3.1.3 (*44*), and all data are available in the Supplementary Materials.

### Analysis on mate search movements of termites

We analyzed movement patterns of termite dealates from before pair formation to after separation. We focused only on a single individual (a female or a male) at each observation, and thus, the obtained data of movement sequences were not paired between sexes. First, we placed a single dealate (either a female or a male) on the experimental arena and observed its movement to search for a mate for 30 min. Then, we added a single mating partner of the other sex from the same colony (note that we did not analyze the movements of added partners in this experiment). If they formed a tandem within 5 min, we observed a movement of the focal individual during tandem running behavior for 10 min. After 10 min, we carefully removed the added partner using an aspirator and again observed how its movement changed from the state before pair formation for 30 min (movies S1 and S2). As we used the confined space (petri dish) for the experiments, the observed movement patterns can be affected by borders and might be biased. However, this did not affect our primary purpose, i.e., the comparison between sexes and between searching schemes. In case a tandem pair was not formed within 5 min, we ceased the trial. Each individual was used only once for data collection. We obtained full behavioral observations of 19 males (R_A_, 5; R_C_, 4; R_D_, 6; R_E_, 4) and 18 females (R_A_, 5; R_C_, 3; R_D_, 6; R_E_, 4) in *R. speratus*, and 23 males (C_A_, 7; C_B_, 4; C_L_, 12) and 23 females (C_A_, 8; C_B_, 3; C_L_, 12) in *C. formosanus*.

We first compared the movement patterns between sexes. We described the movement patterns of termites by move-pause patterns, moving speed, and turning patterns. First, the movements of each individual were discretized into a series of moves and pauses. The distribution of the length of displacements between successive frames (0.2 s) was bimodal, with two peaks around 0 and 3.5 mm for *R. speratus*, and 0 and 4.7 mm for *C. formosanus*. The two peaks can be regarded as representing pauses and moves, respectively. We obtained the thresholds for move/pause as the value representing the second peak multiplied by 0.2 (= 0.70 mm for *R. speratus*; 0.94 mm for *C. formosanus*), where pause was defined as displacement less than or equal to the thresholds (the positions of the values in the histograms were shown in fig. S7). These values were close to our visual discrimination of move and pause during video observations. We examined other threshold values, but it did not affect our conclusions qualitatively. We calculated the duration of pauses in every minute for before encounters (30 min), during tandem running (10 min), and after separation (30 min). The moving speeds were computed by the instantaneous speed when termites were moving. The turning angles were computed as the magnitude of changes in the direction of motion from one frame to the next frame during moving. We also calculated turning angles for the direction changes before and after pauses. Then, the mean values of moving speeds and turning angles were obtained in every minute for before pair formation (30 min), during tandem running (10 min), and after separation (30 min). These three components (duration of pauses, moving speeds, and turning angles) were compared between sexes every minute using Wilcoxon rank sum test with Bonferroni correction for multiple comparison (α = 0.05/70).

As a result of the above analysis, we found that the sexual dimorphism was prominent just after separation in both species (Fig. 2, B and C). We focused on 1 min after separation to explore the difference in movement patterns between before pair formation and after separation. For comparison, we prepared those of the last 1 min (29 to 30 min) before pair formation. We used CRWs to measure differences in movement patterns of termites between searching schemes and between sexes, which accounts for the emergence of angular correlations in animal trajectories coming from local scanning behavior (*10*, *26*). CRW can be described by two parameters: speed and sinuosity. The speed was obtained by the mean of moving speeds. The sinuosity was given by the scale parameter, which was obtained by fitting wrapped Cauchy distributions to turning angle data using maximum likelihood estimation methods. Wrapped Cauchy distribution covers from uniform distribution (scale parameter = 0) to delta distribution (scale parameter = 1) and corresponding movement patterns of Brownian and straight walks, respectively (*4*). We tested the difference of these two parameters between before pair formation and after separation using Wilcoxon signed-rank test, and between sexes using Wilcoxon rank sum test with Bonferroni correction for multiple comparison (α = 0.05/4). Although it is beyond the scope of this study, we also checked if there were colony-level variations in these parameters for each sex and each treatment, using Kruskal-Wallis (*R. speratus*) or Wilcoxon rank sum (*C. formosanus*) test with Bonferroni correction for multiple comparisons (α = 0.05/4). We found a significant between-colony difference only in male *C. formosanus* before pair formation (*P* = 0.006).

In addition, we examined frequency distributions of the durations of moves and pauses for each sex and searching scheme, respectively. In measuring the durations of moves and pauses, we modified the onsets and the endpoints of observation (i.e., the observed period was shorter or longer than 1 min) to avoid the artifact caused by the bound of observation period. Before pair formation, we measured the behavioral sequence from the beginning of the behavior that was performed at 29 min to the end of the behavior that finished before 30 min (i.e., we omitted the behavior that was performed at 30 min). On the other hand, after separation, we measured the behavioral sequence from the behaviors that was performed at 0 min to the end of the behavior that was performed by termites at 1 min. We fitted stretched exponential distributions and truncated power-law distributions with a minimum value of 0.2 s to all data. As our data were digitized in 0.2 s (i.e., frames), we used the methods of maximum likelihood approach for pre-binned datasets as described in the previous studies (*28*, *45*), where we numerically estimated the parameter μ for truncated power-law and the parameters λ and β for stretched exponential distributions that maximized the log likelihood (see Supplementary Text). For truncated power-law distributions, we set maximum values as the maximum value observed in each dataset. Model selection was based on the Akaike weight. We also performed goodness-of-fit test for better fitted models, based on Kolmogorov-Smirnov (KS) statistic, according to Clauset *et al.* (*46*). We generated 2500 datasets, each consisting of the same number of data points as the observed data, from the distribution model we tried to test. After digitizing these generated data in 0.2-s bins (e.g., data point *x* with the value of 0.2 ≤ *x* < 0.4 becomes 0.2), we individually fitted the original distribution model to each dataset and calculated the KS statistic. Then, we simply counted the number of KS statistics larger (i.e., more mismatched) than that obtained by using observed data to obtain the *P* value. This *P* value indicates how likely the observed data are derived from the tested model. For analysis, data were pooled over individuals within each sex and searching scheme to obtain enough sample size.

We also examined the reorientation behavior (i.e., turning behavior after pauses). We first separated the data of turning angles into those after pauses and those during moving to observe the shape of each distribution. From the visual inspection, the distributions of turning angles displayed a single peak around 0 radian in all cases. Therefore, we simply fitted wrapped Cauchy distribution to each of them. We also compared these distributions between after pauses and during movements, as well as between before pair formation and after separation, using the KS test. For this analysis, we pooled the data over individuals within each sex and each scheme to get enough sample sizes. We also examined the relationship between the length of pause duration and the absolute value of turning angles using linear regression for each species.

### Simulations

We developed an individual-based model to examine the efficiency of movement patterns observed in termites. We prepared two searching situations, a periodic boundary condition of size = *L* × *L* and a borderless continuous space where a female and a male initially separated by the distance *d*. The former was to simulate uninformed search before pair formation, and the latter was to simulate reunion search after separation. In each condition, we considered a female and a male walking until encountering another individual of the other sex. When the distance between the centers of the two individuals became smaller than φ, they were regarded to encounter. This φ value is based on the definition of tandem running, with 7 mm for *R. speratus* and 10 mm for *C. formosanus* (see Supplementary Text).

Individuals perform CRW with the parameters of speed and sinuosity, denoted by *v* and ρ, respectively. The speed parameter *v* was obtained as the mean value of the observation data for each sex and search scheme (Table 1), while the sinuosity parameter ρ was obtained as the estimated scale parameter from the data of turning angles during moving (table S1). On the basis of our behavioral analysis, each time step was adjusted to 0.2 s. Thus, each individual moved 0.2*v* mm in each time step. Turning angles followed wrapped Cauchy distribution with scale parameter ρ. After generating a uniform random number *u* (0 < *u* ≤ 1), the turning angles θ were derived from the following equation by applying the inversion method (*10*)

$$\theta =2\text{arcsin}[(\frac{1-\rho^{*}}{1+\rho^{*}})\text{tan}[\pi (u-0.5)]]$$

We initiated the simulation with a random bearing angle that fluctuated according to θ. At each step, the bearing angle was equal to the previous bearing angle plus the deviation θ such that the moving object always kept the previous direction, forming a CRW.

We added move-pause intermittency to the above CRWs to account for pausing behaviors. Observations showed that their distributions of the duration of moves and pauses followed either the truncated power-law or stretched exponential distributions. Thus, in the simulation, the duration of moves *t*_m_ and pauses *t*_p_ can be derived from the following equations (*47*). For truncated power-law distributions

$$t_{m}\text{or}t_{p}={\left(x_{\text{max}}^{1-\mu }-u(x_{\text{max}}^{1-\mu }-x_{\text{min}}^{1-\mu })\right)}^{\frac{1}{1-\mu }}$$

and for stretched exponential distributions

$$t_{m}\text{or}t_{p}={\left(x_{\text{min}}^{\beta }-\frac{1}{\lambda }u\right)}^{\frac{1}{\beta }}$$

where *x*_min_ is the minimum value of the data (0.2) and *x*_max_ is the maximum value of the data (table S2). Values of parameters are shown in table S2. In addition, we also performed simulations by randomly resampling the duration of moves and pauses from observed datasets directly to confirm that our model fitting worked well to describe the stochastic search rules in termites. These results were qualitatively the same with those using the fitted models (comparing Fig. 3 and fig. S8), indicating that our results are reliable even though goodness-of-fits of some datasets were low (table S2). For the initial condition, individuals were assumed to perform moving or pausing, depending on the proportion of pausing times during observations (fig. S4). When individuals finished pausing, they got a new angle from wrapped Cauchy distribution with a scale parameter for reorientation behavior (table S1), like the above method.

We compared the searching efficiency among four possible combinations of movement patterns—the observed sexually dimorphic movement after separation, the observed sexually monomorphic movement before pair formation, and two virtual monomorphic movements with both a female and a male moving like females or males after separation—for each searching condition in each species. Simulations were performed for 180 s (= 900 time steps). We ran 1,000,000 simulations and measured the efficiency as the probability to encounter a mating partner. The simulation program was implemented in Microsoft Visual Studio C++ 2017. The source codes for all simulations are available in the Supplementary Materials.

## Supplementary Material

http://advances.sciencemag.org/cgi/content/full/5/6/eaau6108/DC1

Download PDF

Movie S1

Movie S2

Data file S1

Data file S2

## SUPPLEMENTARY MATERIALS

Supplementary material for this article is available at http://advances.sciencemag.org/cgi/content/full/5/6/eaau6108/DC1 Supplementary Text Fig. S1. Duration of two different phases observed in tandem running. Fig. S2. Moving speeds of termite dealates across different periods in mate search. Fig. S3. Turning angles of termite dealates across different periods in mate search. Fig. S4. Comparison of the proportion of pausing times between sexes and conditions. Fig. S5. Inverse cumulative frequency distribution of the duration of moves and pauses. Fig. S6. Simulated encounter rates. Fig. S7. Histogram of the length of displacements between successive frames (0.2 s). Fig. S8. Simulation results using empirical data resampling to describe move-pause patterns. Table S1. Parameters on turning patterns extracted from turning angles both during moving and after pauses (reorientation). Table S2. Results of model fitting to moving and pausing time data. Movie S1. Sexual dimorphic movements after separation during tandem running in *R. speratus*. Movie S2. Sexual dimorphic movements after separation during tandem running in *C. formosanus*. Data file S1. All location data. Data file S2. Simulation codes. Reference (*48*)
