# Supplementary material for: Adaptive switch to sexually dimorphic movements by partner-seeking termites
Source: Sci Adv. 2019 Jun 19;5(6):eaau6108. doi: 10.1126/sciadv.aau6108 (PMC6584256; doi:10.1126/sciadv.aau6108)
Supplement: http://advances.sciencemag.org/cgi/content/full/5/6/eaau6108/DC1 [file supp_5_6_eaau6108__index.html]

Science Advances | Science Advances

## Supplementary Materials

**The PDF file includes:**

- Supplementary Text
- Fig. S1. Duration of two different phases observed in tandem running.
- Fig. S2. Moving speeds of termite dealates across different periods in mate search.
- Fig. S3. Turning angles of termite dealates across different periods in mate search.
- Fig. S4. Comparison of the proportion of pausing times between sexes and conditions.
- Fig. S5. Inverse cumulative frequency distribution of the duration of moves and pauses.
- Fig. S6. Simulated encounter rates.
- Fig. S7. Histogram of the length of displacements between successive frames (0.2 s).
- Fig. S8. Simulation results using empirical data resampling to describe move-pause patterns.
- Table S1. Parameters on turning patterns extracted from turning angles both during moving and after pauses (reorientation).
- Table S2. Results of model fitting to moving and pausing time data.
- Legends for movies S1 and S2
- Legends for data files S1 and S2
- Reference (*48*)

Download PDF

**Other Supplementary Material for this manuscript includes the following:**

- Movie S1 (.mp4 format). Sexual dimorphic movements after separation during tandem running in *R. speratus*.
- Movie S2 (.mp4 format). Sexual dimorphic movements after separation during tandem running in *C. formosanus*.
- Data file S1 (.zip format). All location data.
- Data file S2 (.cpp format). Simulation codes.

**Files in this Data Supplement:**

- Adobe PDF - aau6108\_SM.pdf
