# Supplementary material for: Adaptive switch to sexually dimorphic movements by partner-seeking termites
Source: Sci Adv. 2019 Jun 19;5(6):eaau6108. doi: 10.1126/sciadv.aau6108 (PMC6584256; doi:10.1126/sciadv.aau6108)
Supplement: Download PDF [file aau6108_SM.pdf]

Science Advances
AAAS

advances.sciencemag.org/cgi/content/full/5/6/eaau6108/DC1

Supplementary Materials for

Adaptive switch to sexually dimorphic movements by partner-seeking termites

Nobuaki Mizumoto* and Shigeto Dobata

*Corresponding author. Email: nobuaki.mzmt@gmail.com

Published 19 June 2019, *Sci. Adv.* **5**, eaau6108 (2019)
DOI: 10.1126/sciadv.aau6108

The PDF file includes:

Supplementary Text
Fig. S1. Duration of two different phases observed in tandem running.
Fig. S2. Moving speeds of termite dealates across different periods in mate search.
Fig. S3. Turning angles of termite dealates across different periods in mate search.
Fig. S4. Comparison of the proportion of pausing times between sexes and conditions.
Fig. S5. Inverse cumulative frequency distribution of the duration of moves and pauses.
Fig. S6. Simulated encounter rates.
Fig. S7. Histogram of the length of displacements between successive frames (0.2 s).
Fig. S8. Simulation results using empirical data resampling to describe move-pause patterns.
Table S1. Parameters on turning patterns extracted from turning angles both during moving and after pauses (reorientation).
Table S2. Results of model fitting to moving and pausing time data.
Legends for movies S1 and S2
Legends for data files S1 and S2
Reference (*48*)

Other Supplementary Material for this manuscript includes the following:

(available at advances.sciencemag.org/cgi/content/full/5/6/eaau6108/DC1)

Movie S1 (.mp4 format). Sexual dimorphic movements after separation during tandem running in *R. speratus*.
Movie S2 (.mp4 format). Sexual dimorphic movements after separation during tandem running in *C. formosanus*.
Data file S1 (.zip format). All location data.
Data file S2 (.cpp format). Simulation codes.

Supplementary Materials

Supplementary Text

Supporting Information for “Adaptive switch to sexually dimorphic movements by partner-seeking termites.”

SI text

Observations of tandem running

We observed a full sequence of tandem running of termites. For observations, we used pairs of individuals that flew on the same day. All pairs were formed by individuals from the same colonies. We placed a female and a male on the experimental arena (*Materials and Methods*) and observed them for 60 minutes. In case that no tandem running was observed in 60 minutes, we excluded the data from the following analysis. In one replicate, as we had a trouble in the video (image got misaligned), we divided the file into two different files for analysis. Totally, we obtained the data of 28 pairs of *R. speratus* (R_A: 3 pairs, R_B: 6 pairs, R_c: 5 pairs, $R_{D, E}$: 7 pairs) and 17 pairs of *C. formosanus* (C_A: 13 pairs, C_B: 4 pairs).

In this study, we defined the tandem running using the following three different criteria: (i) we determined that termites perform tandem running when distance between centroids of a female and a male was less than 7 mm (for *R. speratus*) or 10 mm for (*C. formosanus*) for more than 3.0 seconds. As a female and a male nearly had physical contact during tandem running, these distances were adjusted to slightly exceed the body length of termites including antenna; (ii) when females did not move for more than 30 mm during events of (i), we determined that this period was not the tandem running. This treatment enabled image analysis to distinguish tandem running from the event where the two individuals were just close to each other by chance; (iii) when an angle between moving direction of a female and a male was less than 45 degrees during two separate tandem running events, we defined this period also being in tandem running. This is because a female and a male sometimes perform tandem running without contact for a while when the moving speed of a female increased. Based on these three criteria, we measured the duration of tandem running, the distance between a female and a male during reunion search, and the time required for a female and a male to encounter again. We analyzed the duration of tandem running and the time required for reunion by generating Kaplan-Meier survival curves. The Kaplan-Meier survival curve is defined as the probability of an event not happening in a given time interval (*48*). Events that were disrupted because of the end of observation were right-censored. Survival analysis was conducted using the ‘survival’ package in R.

As a result, we observed 283 events of separation in 26 of 28 pairs in *R. speratus*, while 58 events in 15 of 17 pairs in *C. formosanus*. Tandem running was maintained longer in *C. formosanus* than in *R. speratus* (log-rank test, $\chi^2_1 = 16.4$, $P < 0.0001$; fig. S1*A*). When a mating pair got separated, it took longer time for reunion in *C. formosanus* than in *R. speratus* (log-rank test, $\chi^2_1 = 17.4$, $P < 0.0001$; fig. S1*B*). During reunion search, a male and a female were separated in longer distance in *C. formosanus* than in *R. speratus*, where the mode value of the distribution of separated distance was 16.09 mm for *R. speratus* and 22.97 mm for *C. formosanus*. All reunion search were completed in 3.4–124.6 sec. in *R. speratus* (n = 282), and in 3.4–151.8 sec. in *C. formosanus* (n = 56) (fig. S1*C*, *D*).

The maximum likelihood methods

In this study, moving/pausing patterns of termites were described by either truncated power-law distribution or stretched exponential distribution. The probability distribution functions of these distributions are defined as follows (*46*):

Truncated power-law: $p(x) = \dfrac{\mu - 1}{x_{\min}^{1-\mu} - x_{\max}^{1-\mu}} x^{-\mu}$ and

Stretched exponential: $p(x) = Cx^{\beta-1}e^{-\lambda x^{\beta}}$; $C = \beta\lambda e^{\lambda x_{\min}^{\beta}}$.

Because of the pre-binned nature of our data (i.e., frames), we obtained log-likelihood functions according to the Edwards et al. (2007). We used the same equation with Edwards et al. (2007) for truncated power-law, and we obtained the equation for stretched exponential accordingly. The probability that a single data value locates within bin *j* given the parameters λ and β is

$$
\begin{aligned}
P(\text{being in bin } j \,|\, \beta, \lambda) &= \int_{x_{\min}+(j-1)w}^{x_{\min}+jw} Cx^{\beta-1}e^{-\lambda x^{\beta}}\,dx \\
&= \left[-\frac{Ce^{-\lambda x^{\beta}}}{\beta\lambda} \right]_{x_{\min}+(j-1)w}^{x_{\min}+jw} \\
&= -\frac{C}{\beta\lambda}\left[e^{-\lambda(x_{\min}+jw)^{\beta}} - e^{-\lambda(x_{\min}+(j-1)w)^{\beta}} \right] \\
&= e^{\lambda x_{\min}^{\beta}}\left[e^{-\lambda(x_{\min}+(j-1)w)^{\beta}} - e^{-\lambda(x_{\min}+jw)^{\beta}} \right]
\end{aligned}
$$

where *w* indicates the bin length (= 0.2). Thus, with *n* data points, the log-likelihood function is

$$
\begin{aligned}
l(\beta, \lambda \,|\, \text{data}) &= \sum d_j P(\text{being in bin } j \,|\, \beta, \lambda) \\
&= n\lambda x_{\min}^{\beta} + \sum d_j \log\left[e^{-\lambda(x_{\min}+(j-1)w)^{\beta}} - e^{-\lambda(x_{\min}+jw)^{\beta}} \right]
\end{aligned}
$$

which was used to find maximum likelihood estimates.

The role of pausing behavior in reunion search

In the movements of termites, one remarkable characteristic was the pausing behavior observed in females during reunion search. To investigate the advantage of pausing behavior in reunion search, we added the analysis for the encounter rates with two types of females with extreme strategies: without pausing and only pausing. The former moves with the parameter of females after separation (Table 1) but does not pause; the latter never moves. Other settings were the same as the simulation conditions in Fig. 3. We ran 1,000,000 simulations and measured the efficiency as the probability to encounter with a mating partner. As a result, we found that the only pausing strategy achieved the highest encounter rates in reunion search with the conditions of both *R. speratus* and *C. formosanus*, while the only moving strategy had even lower encounter rates than the observed intermittent strategy (fig. S6). Thus, in reunion search, the pausing behavior significantly contributed to the encounter rates.

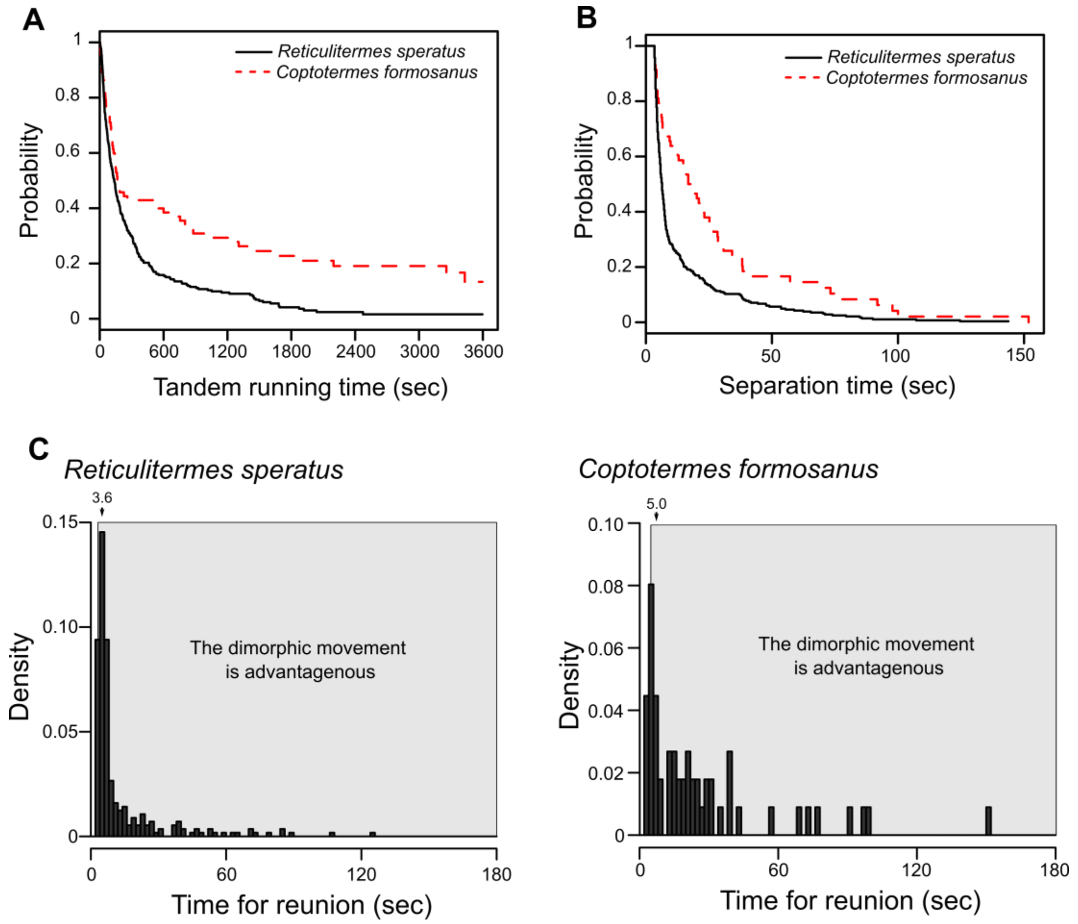

Fig. S1. Duration of two different phases observed in tandem running. (**A**) Continuation time of tandem running until a pair got separated and lost (sec). Tandem running of *Coptotermes formosanus* was likely to continue longer than that of *Reticulitermes speratus* (log-rank test, $\chi^2_1 = 16.4$, $P < 0.0001$). (**B**) Time required for a separated pair to reunion (sec). Pairs of *C. formosanus* required longer time to reunion than those of *R. speratus* when they were separated (log-rank test, $\chi^2_1 = 17.4$, $P < 0.0001$). (**C**) The distribution of time required to reunion. The shade areas indicate the search time in which observed sexually dimorphic movements achieved higher efficiency than the observed sexually monomorphic movements (Fig. 3).

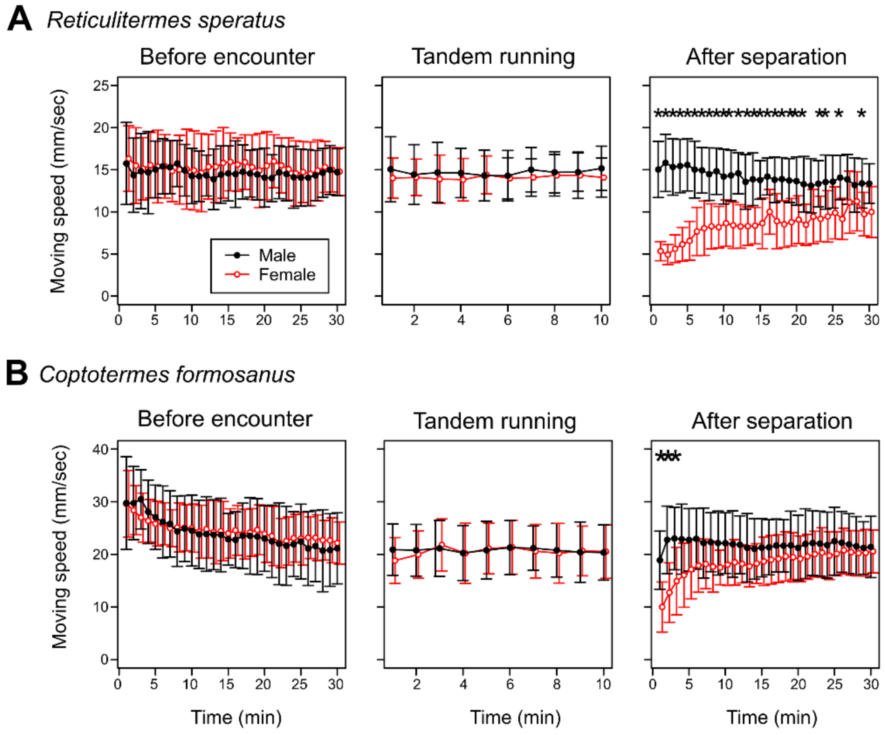

Fig. S2. Moving speeds of termite dealates across different periods in mate search. We first observed searching behavior of males and females before pair-formation. Then we added single mating partner of the other sex to observe their movements during tandem running. Finally we carefully removed the partner using an aspirator and observed how they change their searching behaviors. (**A, B**) Comparison of moving speeds between sexes in (**A**) *R. speratus* and (**B**) *Coptotermes formosanus*. Females of both species specially decrease their movement speeds immediately after separation, where *R. speratus* showed distinct sexual dimorphism. Points with bars represent mean values with standard deviations. * indicates the significant difference (Wilcoxon rank-sum test with Bonferroni corrections, $P < 0.05/70$).

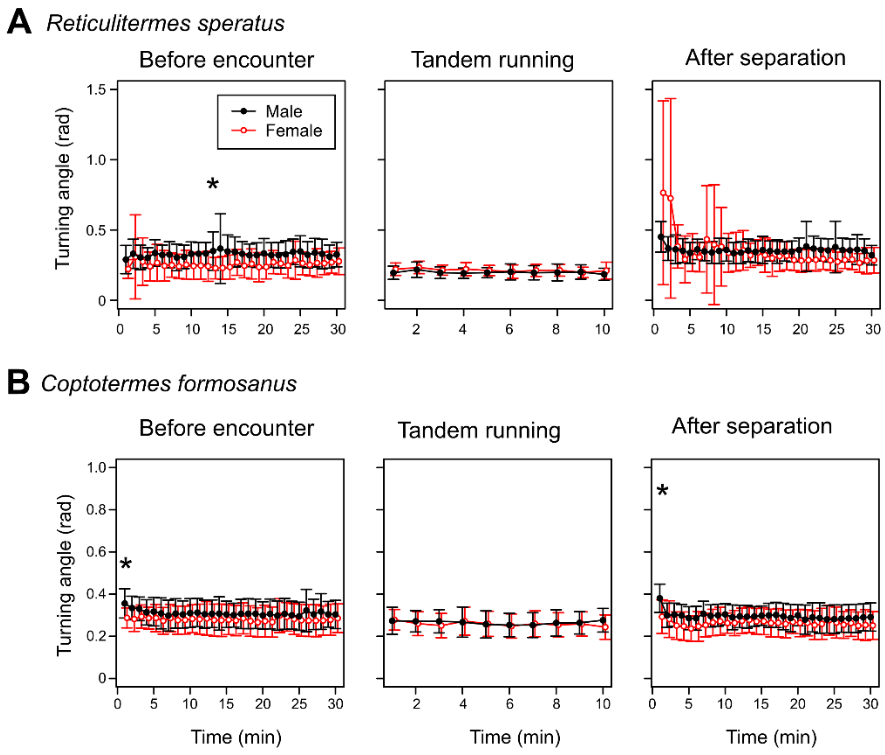

Fig. S3. Turning angles of termite dealates across different periods in mate search. We first observed searching behavior of males and females before pair-formation. Then we added single mating partner of the other sex to observe their movements during tandem running. Finally we carefully removed the partner using an aspirator and observed how they change their searching behaviors. (**A, B**) Comparison of turning angles between sexes in (**A**) *R. speratus* and (**B**) *Coptotermes formosanus*. Points with bars represent mean values with standard deviations. * indicates the significant difference (Wilcoxon rank-sum test with Bonferroni corrections, $P < 0.05/70$).

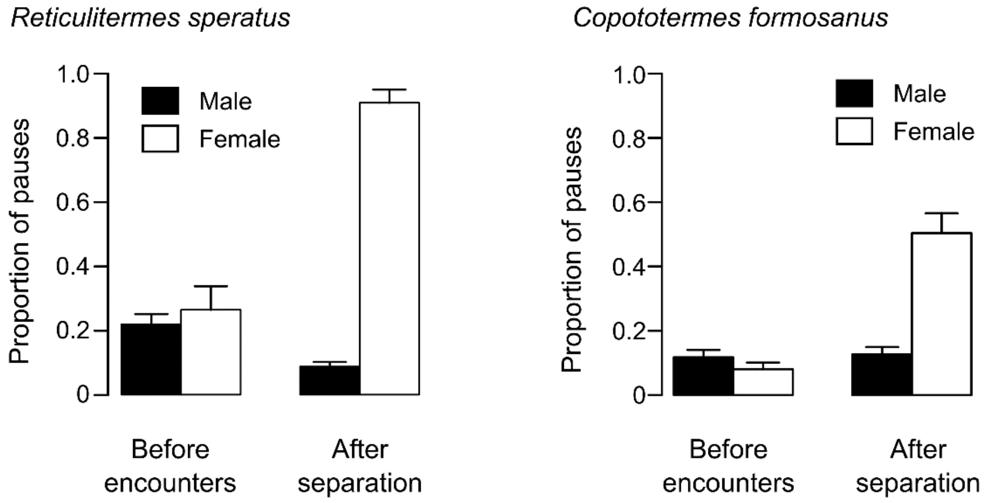

Fig. S4. Comparison of the proportion of pausing times between sexes and conditions. In both species, females paused more than half of periods in average.

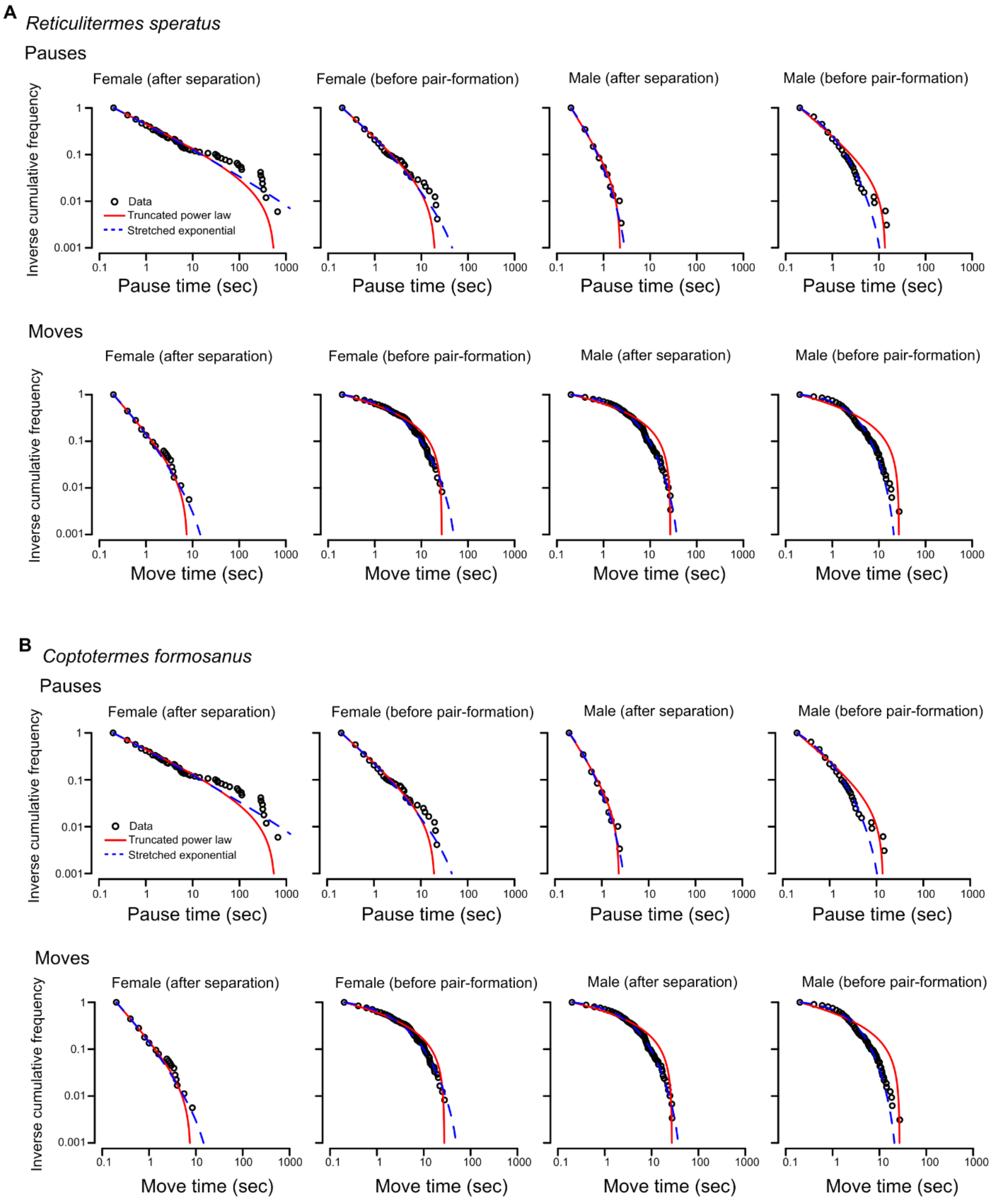

Fig. S5. Inverse cumulative frequency distribution of the duration of moves and pauses. (**A**) *R. speratus* and (**B**) *C. formosanus*. Dots are observations, red and blue dashed lines are truncated power-law and stretched exponential distributions, respectively, fitted to the data. As each flame was sampled every 0.2 seconds, the durations of moves and pauses are in increment of 0.2 seconds (≥ 0.2 seconds).

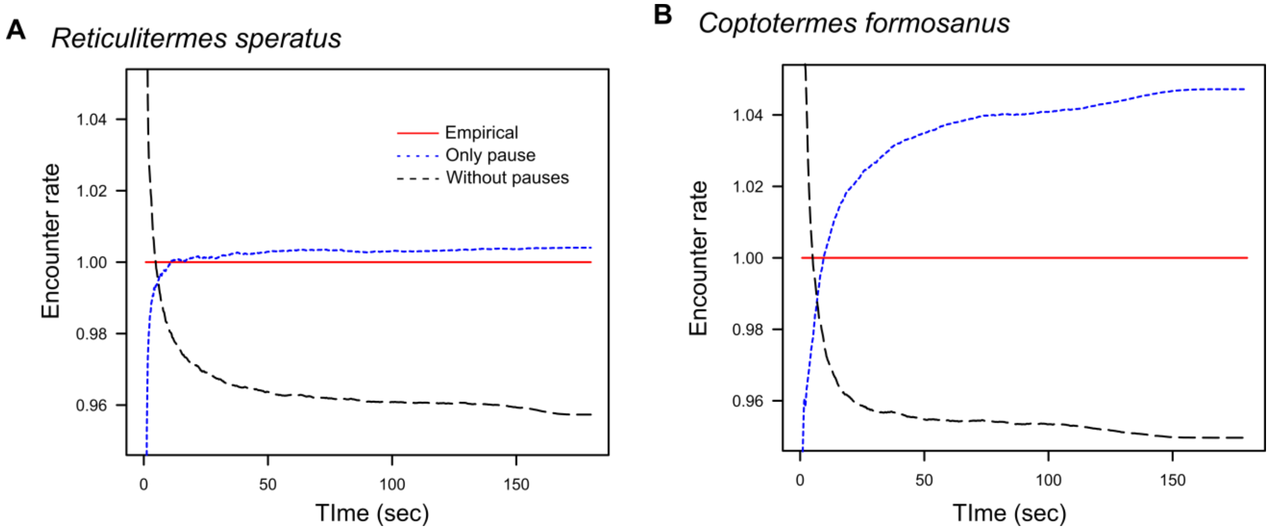

Fig. S6. Simulated encounter rates. The encounter rates of the females with observed strategy, only pausing strategy and without pausing strategy in reunion search. The efficiency is computed as the encounter rates in 1,000,000 trials, where relative efficiencies to the observed strategy were described. (A) The results with the condition of *Reticulitermes speratus*, and (B) *Coptotermes formosanus*.

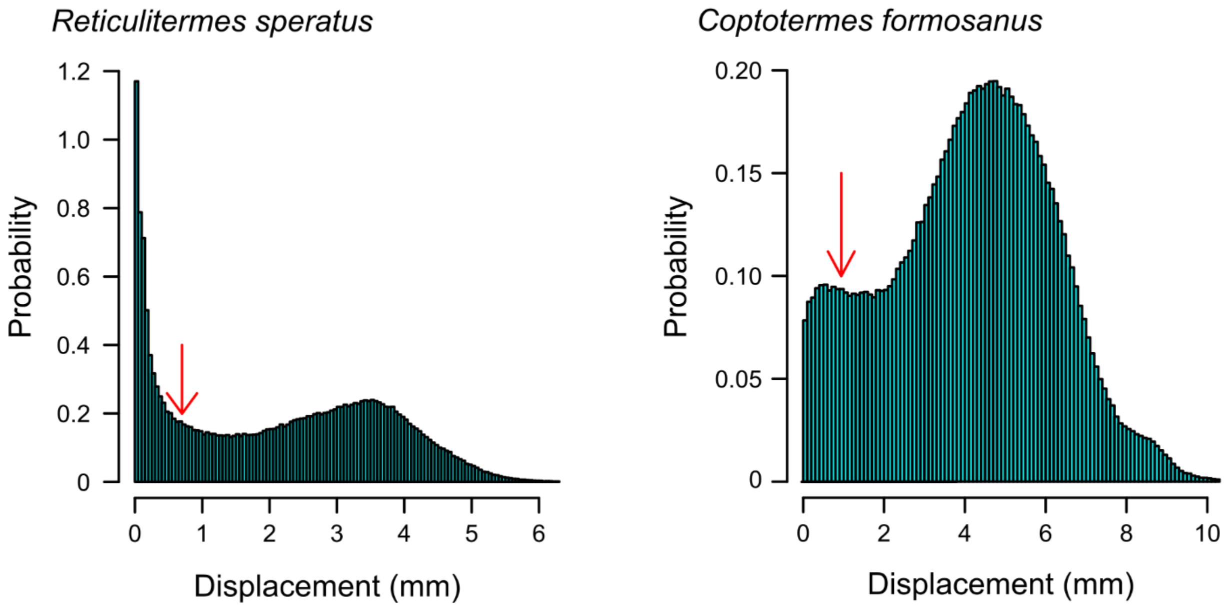

Fig. S7. Histogram of the length of displacements between successive frames (0.2 s). That is the distributions of length of position changes in 0.2 seconds (between two frames). Each histogram was produced by pooling the data of all individuals. Red arrows indicate the threshold values for move/pause (= 0.70 mm for *R. speratus*; 0.94 mm for *C. formosanus*).

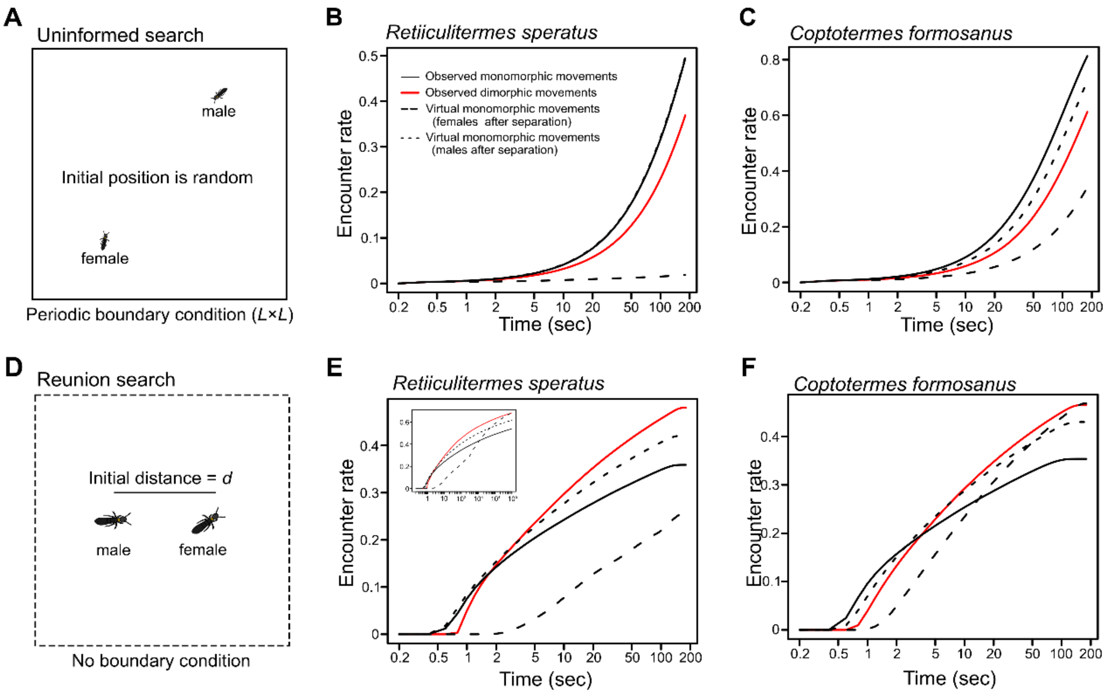

Fig. S8. Simulation results using empirical data resampling to describe move-pause patterns. (A) Assumed search conditions before pair-formation (uninformed search). (B, C) Searching efficiency under uninformed search conditions for (B) *Reticulitermes speratus* and (C) *Coptotermes formosanus*. (D) Assumed search conditions after a pair gets separated (reunion search). (E, F) Searching efficiency of observed movement patterns under reunion search conditions for (E) *R. speratus* and (F) *C. formosanus*. The results were obtained from means of 1,000,000 simulations. Parameters other than move-pause patterns are same with Fig. 3.

Table S1. Parameters on turning patterns extracted from turning angles both during moving and after pauses (reorientation).

A *Reticulitermes speratus*

Male	**Sinuosity**		**Kolmogorov–Smirnov test**	
	Moving	Reorientation	*D*	*P* value
Before	0.86	0.69	0.227	<0.001
After	0.78	0.68	0.122	0.001

Female	**Sinuosity**		**Kolmogorov–Smirnov test**	
	Moving	Reorientation	*D*	*P* value
Before	0.87	0.68	0.219	<0.001
After	0.84	0.73	0.180	0.003

B *Coptotermes formosanus*

Male	**Sinuosity**		**Kolmogorov–Smirnov test**	
	Moving	Reorientation	*D*	*P* value
Before	0.84	0.75	0.141	<0.001
After	0.80	0.67	0.119	<0.001

Female	**Sinuosity**		**Kolmogorov–Smirnov test**	
	Moving	Reorientation	*D*	*P* value
Before	0.86	0.68	0.272	<0.001
After	0.85	0.74	0.173	<0.001

The sinuosity corresponds to the scale parameter of wrapped Cauchy distributions, covering from 0 (most sinuous) to 1 (straight motion). Kolmogorov–Smirnov tests were performed to compare the distributions.

Table S2. Results of model fitting to moving and pausing time data.

Species	Move/Pause	Sex	Scheme	# of data	Min. data	Max data	μ (TP)	λ (SE)	β (SE)	TP wAIC	SE wAIC	Judge	GOF
R. speratus													
	Pause	Female	After	169	0.2	661	**1.488**	27.567	0.019	0.976	0.024	TP	0.381
			Before	243	0.2	22	**1.894**	10.203	0.099	0.959	0.041	TP	0.786
		Male	After	297	0.2	2.4	**2.563**	6.601	0.378	0.897	0.103	TP	0.734
			Before	326	0.2	14.6	1.819	**2.795**	**0.431**	0.000	1.000	SE	0.820
	Move	Female	After	178	0.2	8.4	**2.145**	11.037	0.122	0.926	0.074	TP	0.467
			Before	243	0.2	27.8	**1.120**	0.675	0.580	0.567	0.433	TP	0.736
		Male	After	296	0.2	27.4	1.065	**0.499**	**0.705**	0.000	1.000	SE	0.051
			Before	322	0.2	27.2	1.131	**0.478**	**0.851**	0.000	1.000	SE	0.445
C. formosanus													
	Pause	Female	After	400	0.2	41.4	**1.791**	26.524	0.032	0.997	0.003	TP	0.030
			Before	196	0.2	6	**2.215**	47.569	0.028	0.975	0.025	TP	0.422
		Male	After	349	0.2	6.6	**2.204**	7.541	0.201	0.948	0.052	TP	0.485
			Before	253	0.2	7.6	**1.965**	5.909	0.213	0.984	0.016	TP	0.543
	Move	Female	After	394	0.2	49.2	**1.666**	6.295	0.122	0.997	0.003	TP	0.129
			Before	200	0.2	483.8	1.236	**0.527**	**0.501**	0.000	1.000	SE	0.087
		Male	After	347	0.2	64.2	**1.361**	1.525	0.325	0.775	0.225	TP	0.232
			Before	254	0.2	328.2	1.323	**0.948**	**0.387**	0.000	1.000	SE	0.010

TP is truncated power-law, SE is stretched exponential, and GOF is goodness-of-fit. Parameters μ, λ and β are estimated by maximum likelihood methods. The goodness-of-fits were based on the Kolmogorov-Smirnov statistic (*46*), which indicates p value of how likely is that the data comes from the fitted model. Values larger than 0.1 suggests that the data should be from the model. Parameters used in the simulations are shown in bold.

Legends for supplemental Movies

Movie S1. Sexual dimorphic movements after separation during tandem running in *R. speratus*. A male or a female is removed by using an aspirator.

Movie S2. Sexual dimorphic movements after separation during tandem running in *C. formosanus*. A male or a female is removed by using an aspirator.

Data file S1. All location data.

Data file S2. Simulation codes.
